# Exploring the relationship between ultrasound parameters and muscle strength in older adults: a meta-analysis of sarcopenia-related exercise performance

**DOI:** 10.3389/fmed.2024.1429530

**Published:** 2024-09-27

**Authors:** Han Yuan, Maeng-Kyu Kim

**Affiliations:** ^1^Department of Physical Education, Graduate School, Kyungpook National University, Daegu, Republic of Korea; ^2^Sports Science Research Institute, Kyungpook National University, Daegu, Republic of Korea

**Keywords:** ultrasonography, sarcopenia, muscle strength, physical functional performance, older adult

## Abstract

**Introduction:**

Ultrasound (US) imaging has emerged as a promising tool for assessing age-related muscle changes. This meta-analysis aimed to comprehensively evaluate the associations between US parameters and muscle strength, as well as sarcopenia-related functional performance in older adults by integrating data from multiple studies.

**Methods:**

A systematic literature search was conducted in PubMed, Web of Science, and Embase until June 2023. Studies reporting Pearson’s correlation coefficients between US parameters [echo intensity (EI), muscle thickness (MT), cross-sectional area (CSA), pinnations angle (PA), fascicle length (FL)] and measures of muscle strength or physical performance in older adults were included. Effect sizes were pooled using a random-effects model and presented in forest plots. Heterogeneity was assessed using *I*^2^, and publication bias was evaluated using Egger’s test.

**Results:**

Twenty-eight studies met the inclusion criteria. Meta-analysis revealed moderate to strong correlations between EI, MT, and CSA with muscle strength. However, no significant associations were found between US parameters and gait speed. For chair stand tests, the strength of associations varied by test type, with weak correlations observed between echo intensity and muscle thickness with sit-to-stand tests. US parameters did not exhibit significant correlations with the Timed Up and Go test.

**Conclusion:**

Ultrasonographic measurements of echo intensity (EI) and muscle thickness (MT) demonstrated moderate to strong correlations with muscle strength and functional assessments related to sarcopenia. To enhance the accuracy of sarcopenia diagnosis and the effectiveness of management strategies, there is a need for larger, longitudinal studies that evaluate a comprehensive range of ultrasonographic parameters.

**Systematic review registration:**

https://inplasy.com, identifier INPLASY202410086.

## 1 Introduction

Sarcopenia, characterized by the age-related loss of muscle mass, strength, and function, has emerged as a significant clinical concern impacting the health and wellbeing of older adults worldwide (1). The European Working Group on Sarcopenia in Older People (EWGSOP) reports a prevalence ranging from 9.9 to 40.4% among community-dwelling older individuals, (2) highlighting the urgency for effective diagnostic and therapeutic strategies. This geriatric syndrome is associated with an increased risk of adverse outcomes, such as falls, fractures, physical disability, and a diminished quality of life (3, 4). In response to this clinical challenge, researchers have increasingly explored the potential of ultrasound (US) imaging as a non-invasive, cost-effective, and readily accessible tool for assessing muscle quality and morphology in older adults.

It is important to note that while there is a global recognition of sarcopenia as a significant health issue, there are notable differences in its definition and diagnostic criteria across regions (1). The EWGSOP and the Asian Working Group for Sarcopenia (AWGS) have both established guidelines, but with some distinct features. The EWGSOP emphasizes muscle strength as the primary parameter for identifying sarcopenia, followed by muscle quantity or quality, and physical performance (2). In contrast, the AWGS recommends simultaneous measurement of both muscle strength and muscle mass as the core diagnostic elements, with physical performance as an indicator of severity (5). These differences reflect the potential influence of ethnic and cultural factors on sarcopenia manifestation and highlight the need for population-specific approaches in its assessment and management (6).

Given the complexity and variability in sarcopenia definitions and manifestations across populations, there is a pressing need for reliable, accessible, and versatile diagnostic tools. In response to this clinical challenge, researchers have increasingly explored the potential of US imaging as a non-invasive, cost-effective, and readily accessible tool for assessing muscle quality and morphology in older adults. US imaging offers a non-invasive, cost-effective, tolerable, rapid, real-time, and portable method for evaluating muscle characteristics, rendering it a valuable asset in the clinical assessment and management of sarcopenia. Its ability to accurately and reliably measure muscle quality, with high repeatability, has been well-established (4). Commonly utilized US parameters, including muscle thickness (MT), cross-sectional area (CSA), pennation angle (PA), fascicle length (FL), and echo intensity (EI), provide insights into muscle morphology, structure, and quality, finding extensive application in sarcopenia research (6, 7). Notably, the updated EWGSOP consensus recommends US as an effective and reliable tool for measuring muscle quality, affirming its utility in sarcopenia diagnosis (2).

Our previous meta-analysis, (8) focused solely on the association between EI and muscle strength/functional performance. The present study aims to conduct a comprehensive examination of multiple US parameters in relation to sarcopenia-related outcomes in older adults. Unlike most previous studies that explored specific US parameters and muscle strength or functional performance separately, this review systematically investigates the collective associations across various US measurements. By combining data across studies through meta-analysis, we aim to provide a quantitative synthesis of the correlations, offering more robust evidence to guide the clinical application of US in sarcopenia assessment and management.

Previous studies have indicated a potential correlation between US measurements and muscle function. For instance, quadriceps MT (*r* = 0.41) (9) and CSA (*r* = 0.78) (10) have demonstrated correlated with knee extension isometric strength. Additionally, quadriceps MT (*r* = 0.52) and EI (−0.33) have shown correlations with handgrip strength in older adults (11). Moreover, evidence indicates significant associations between US parameters and sarcopenia-related exercise performance. Studies identified negative correlations between lower limb muscle FL (*r* = −0.29) and PA (*r* = −0.30) with gait speed. (12) Furthermore, the EI of the vastus lateralis muscle and Timed Up and Go (TUG) (*r* = 0.48), (13) as well as the 5 times sit-to-stand test (5TSTS) (*r* = 0.36) (14), demonstrate significant associations. These studies strongly suggest that structural parameters obtained through US may be associated with muscle strength and functional performance in older adults. However, the findings are limited by the individual study designs, sample sizes, and population characteristics. A meta-analytic approach is needed to provide a more comprehensive and reliable understanding by quantitatively synthesizing and consolidating these associations across multiple studies.

Therefore, this meta-analysis aims to comprehensively examine the associations between US parameters, muscle strength, and sarcopenia-related exercise performance in older adults by synthesizing data across studies. The findings will guide clinical application of US assessments for evaluating muscle health and function, facilitating personalized sarcopenia management to mitigate its impacts and promote healthier aging.

## 2 Materials and methods

This review was conducted in accordance with the Preferred Reporting Items for Systematic Reviews and Meta-Analysis (PRISMA) guidelines (15). Additionally, the review has been registered on Inplasy.com (INPLASY202410086).

### 2.1 Search strategy

A systematic search strategy was employed using Boolean operators on several databases including PubMed, Web of Science, and Embase up until June 2023. Keywords and Boolean operators were modified according to each database’s search strategy, and searches were restricted to studies involving humans, written in English, and reported in peer-reviewed journals. The search strategy is presented in Supplementary Table 1.

### 2.2 Selection criteria

Inclusion criteria Studies must meet the following criteria.

(a) Participants were healthy community residents aged 60 years or older without major neurological and musculoskeletal disorders (b) Muscle mass testing using US and reporting at least one direct assessment of muscle strength or Sarcopenia-related exercise performance (c) Observational studies, including cross-sectional studies, cohort studies, and few case-control (d) Published studies (English).

Articles were excluded if: (a) The participant was currently on medication or had an injury that limited physical activity and independence in daily living (b) The study was conducted in an animal model (c) Received interventions other than usual care or placebo and studies used RCTs experiment (d) The result is partially unable to extract the correlation coefficient (e) Reviews, abstracts, case reports or duplicate published articles (f) Non-English articles.

The selection process was carried out by two independent researchers who screened titles and abstracts of all studies based on the inclusion and exclusion criteria, then reviewed the full text of the remaining studies. Any disagreements were resolved through discussion.

### 2.3 Data extraction

The data extraction process involved coding for author information, year of publication, and population characteristics (sample size, sex, and mean age). The correlation coefficient *r* or standardized beta coefficient between US parameters and two continuous muscle strength or physical function variables were extracted. The test modality/results in the assessment of muscle strength and Sarcopenia-related exercise performance were also coded. Muscle strength was categorized into lower extremity maximum strength (i.e., maximal voluntary force/torque of the force-/torque-time curve [MVC]), explosive force (i.e., rate of force/torque development [RFD/RTD]), handgrip strength (i.e., assessed with a handheld dynamometer [HGS]), while exercise performance indicators was divided into gait speed and mobility. Gait speed (e.g., usual gait speed [UGS] and maximum gait speed [MGS]), chair stand test [e.g., 30-s sit-to-stand test (30SS), 5 times sit-to-stand test (5TSTS), and Timed Up and Go (TUG) test, respectively] were used to classify physical function. If no correlation results were reported, the authors were contacted to obtain the missing information. If the author did not respond, the study was excluded.

### 2.4 Data quality

The risk of bias in the included studies was assessed using the Joanna Briggs Institute (JBI) Analytic Cross-Sectional Study Quality Checklist (Supplementary Table 2). The methodological quality of the selected studies was evaluated based on eight items that assessed inclusion criteria, study participants and setting, criteria for condition measurement, validity and reliability of exposure and outcome measures, confounding factors and resolution strategies, and statistical analysis. Two authors evaluated each item, which was rated as “yes,” “no,” “unclear,” or “not applicable.”

### 2.5 Statistical analysis

The meta-analysis was conducted using Comprehensive Meta-Analysis (CMA) software, version 3.3.070, to analyze the Pearson Product Moment correlation coefficients (*R*-value) obtained from the included studies. The *R*-values were converted into normally distributed variables (z-transformed *Rz*-value) using Fisher’s z transformation (16). The conversion formula is:

$$r_{z}=0.5\,\left[l\,n\,\left(1+r\right)-l\,n\,\left(1-r\right)\right]$$

where ln is the natural logarithm.

The beta coefficient (β) is converted to a value of *r* using the following formula (17).

$$r=0.98\text{β}+0.5\text{γ}\{\begin{array}{c} \text{if} \end{array}\begin{array}{cc} & \begin{array}{cc} & \begin{array}{c} \beta \geq 0,\gamma =1;\\ \beta <0,\gamma =0. \end{array} \end{array} \end{array}$$

The weights of the study were calculated based on the standard errors (SE). The calculation formula is:

$$S\,E=\frac{1}{\sqrt{\left(N-3\right)}}$$

where *n* is the sample size.

The method of inverse variance using random-effect models was chosen, and meta-analysis of the transformed *r* values was then conducted. To interpret the results, pooled *r*_*z*_ values were retransformed to *r* values with inverse Fisher *z* transformation:

$$r=\left(\frac{e^{2\,r\,z}-1}{e^{2\,r\,z}+1}\right)$$

where e is approximately equal to 2.718 and *r*_*z*_ is Fisher-z transformed *r* value (17).

A random-effects model was selected for the meta-analysis.

Correlations (positive or negative) were classified as small (*r* < 0.3), medium (0.3 ≤ *r* ≤ 0.5), or large (*r* > 0.5) (18). Forest plots were used to display studies with 95% confidence intervals and the combined coefficients. The *r z* values were reverse converted to *r* values to classify and interpret the relevant sizes. The heterogeneity of the results between studies was evaluated using the *I*^2^ index, where *I*^2^ ≤ 25% was considered to indicate low heterogeneity, 25% < *I*^2^ < 75% was considered to indicate moderate heterogeneity, and *I*^2^ ≥ 75% was considered to indicate high heterogeneity (19). Finally, to address the possibility of publication bias, we examined funnel plots and used Begg and Mazumdar rank correlations. The Trim and Fill procedure (20) was applied if evidence of publication bias was noted.

## 3 Results

### 3.1 Search characteristics

**FIGURE 1:**
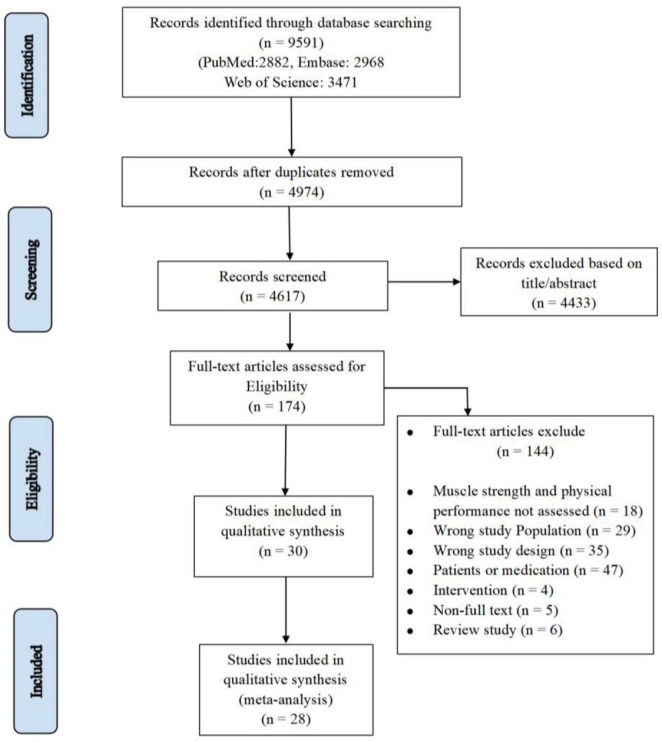
Flow chart selection process.

During the initial database search until June 2023, a total of 9,591 articles were retrieved. After removing duplicates (*n* = 4,974) and excluding 4,617 articles based on title/abstract, 174 articles remained and were assessed for eligibility. Eventually, 28 articles were included in the meta-analysis (Figure 1). A total of 3,599 individuals were included in this review, and the mean age was 73.3 ± 4.6. Sample sizes ranged from 12 to 1,239. Supplementary Table 3 details the baseline characteristics of the included studies.

### 3.2 Association between US parameters and muscle strength

#### 3.2.1 Maximal strength

Fifteen studies (involving 1,861 participants) analyzed the association between EI and maximal strength in older persons with healthy issues (9, 11, 14, 21–32). The results detected a significant strong correlation negative between EI and maximum strength (*r* = −0.56, 95% CI: −0.75 to −0.29, *P* < 0.001, *I*^2^ = 38.92). There was no indication of publication bias (*t* = 1.22, *p* = 0.24; Supplementary Figure 1A).

Thirteen studies (involving 2,836 participant) analyzed the association between MT and maximal strength in healthy older persons (9, 11, 14, 21–26, 28, 30, 32, 33) and a significant moderate correlation was detected between MT and maximal strength (*r* = 0.43, 95% CI: 0.35 to 0.50, *P* < 0.001, *I*^2^ = 59.20). Given indication of publication bias (*t* = 4.58, *p* < 0.01; Supplementary Figure 1B), the Trim and Fill procedure was applied, yielding a mean effect size of 0.47 (95% CI = 1.13 to 3.22).

**FIGURE 2:**
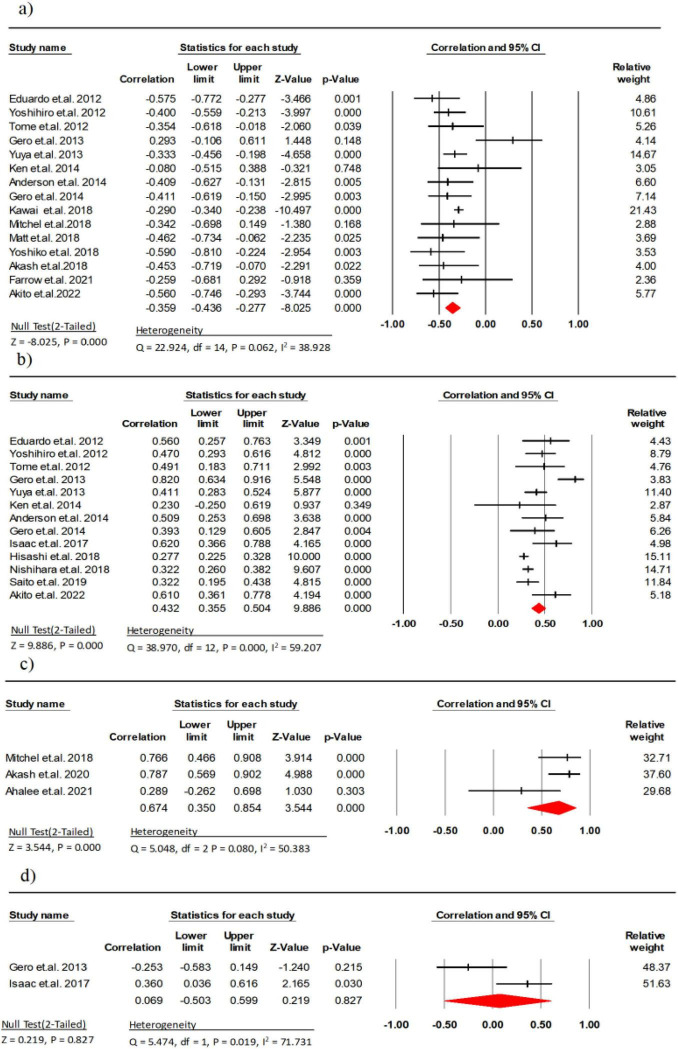
Associations (*r*_*z*_ values) between maximal strength and **(a)** echo intensity, **(b)** muscle thickness, **(c)** cross-sectional area, **(d)** pennation angle. CI, confidence interval; df, degrees of freedom.

Three research studies (comprising 58 participants) examined the link between CSA and maximal strength (10, 27, 31). The results indicated a significant strong correlation between CSA and maximal strength (*r* = −0.67, 95% CI: 0.35 to 0.85, *P* < 0.001, *I*^2^ = 50.38). Two research studies (comprising 62 participants) examined the link between PA and maximal strength (23, 33). The results indicated a weak correlation between PA and maximal strength (*r* = 0.06, 95% CI: −0.50 to 0.59, *P* = 0.827, *I*^2^ = 71.73) (Figure 2). There was also no indication of publication bias (*t* = 1.48, *p* = 0.37; Supplementary Figure 1C).

#### 3.2.2 Explosive power

Two studies (involving 245 participant) analyzed the association between EI and explosive power in healthy older persons (9, 34) and a significant moderate negative correlation was detected between EI and explosive power (*r* = −0.47, 95% CI: −0.88 to 0.35, *P* = 0.256, *I*^2^ = 86.68).

**FIGURE 3:**
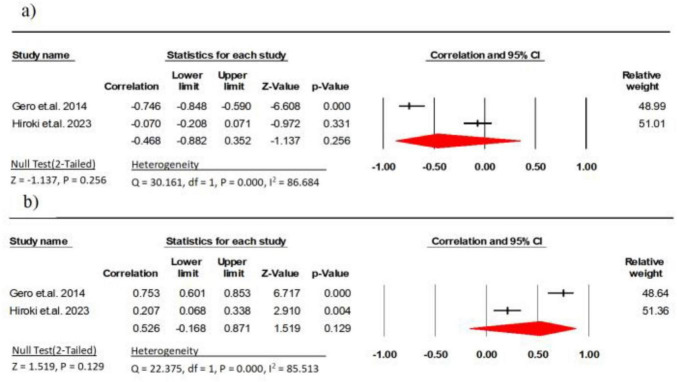
Associations (*r*_*z*_ values) between explosive power and **(a)** echo intensity, **(b)** muscle thickness. CI, confidence interval; df, degrees of freedom.

Two research studies (comprising 245 participants) examined the link between MT and explosive power (9, 34). The results indicated a strong correlation between MT and explosive power (*r* = 0.53, 95% CI: −0.16 to 0.87, *P* = 0.129, *I*^2^ = 85.51) (Figure 3).

#### 3.2.3 Handgrip strength

Three research studies (comprising 199 participants) examined the link between EI and handgrip strength (11, 29, 35). The results indicated a moderate negative correlation between EI and handgrip strength (*r* = −0.32, 95% CI: −0.44 to −0.19, *P* < 0.001, *I*^2^ = 0.000). There was no indication of publication bias (*t* = 2.98, *p* = 0.20; Supplementary Figure 2A).

**FIGURE 4:**
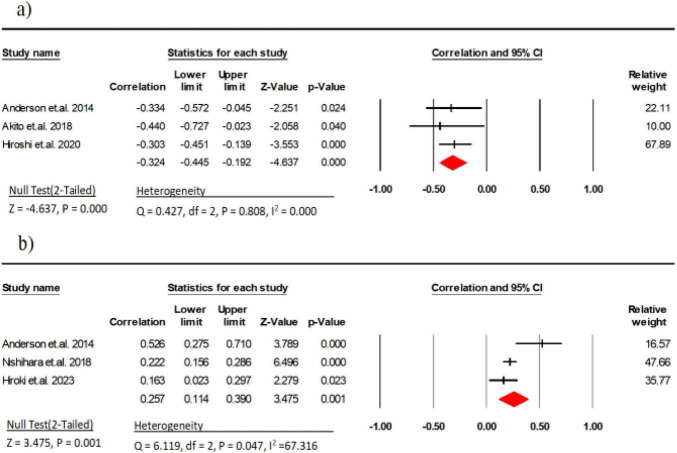
Associations (*r*_*z*_ values) between handgrip strength and **(a)** echo intensity, **(b)** Muscle thickness. CI, confidence interval; df, degrees of freedom.

Three research studies (comprising 1,071 participants) examined the link between MT and explosive power (11, 34, 36). The results indicated a weak correlation between MT and explosive power (*r* = 0.25, 95% CI: 0.11 to 0.39, *P* = 0.001, *I*^2^ = 67.31) (Figure 4). There was no indication of publication bias (*t* = 0.80, *p* = 0.56; Supplementary Figure 2B).

### 3.3 Association between US parameters and physical function

#### 3.3.1 Gait speed

**FIGURE 5:**
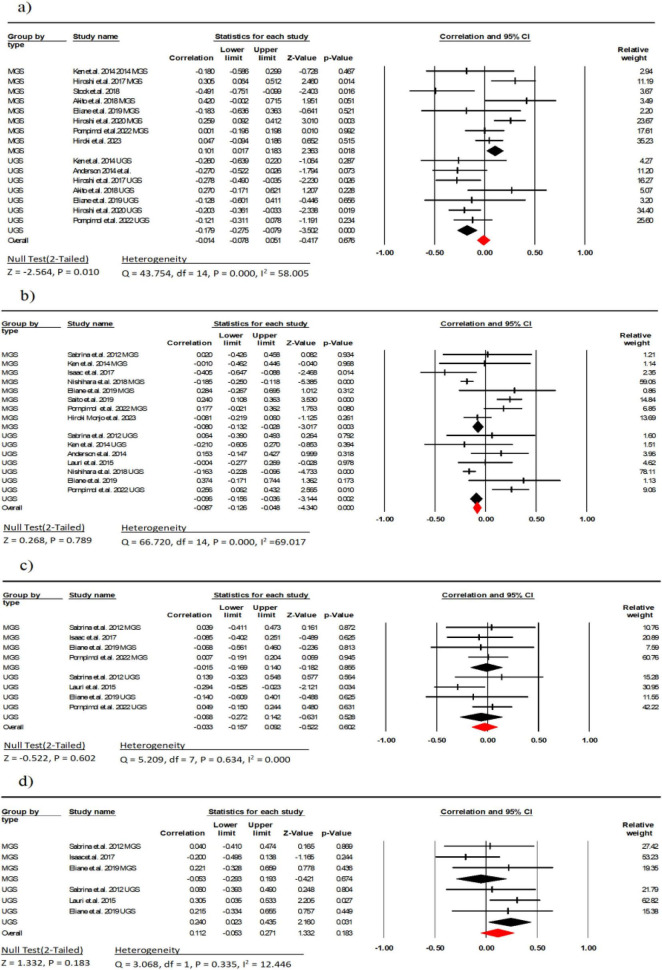
Associations (*r*_*z*_ values) between Gait speed and **(a)** echo intensity, **(b)** Muscle thickness; **(c)** cross-sectional area, **(d)** pennation angle. MGS, maximal gait speed; UGS, usual gait speed; CI, confidence interval; df, degrees of freedom.

Nine studies (involving 614 participants) investigated the association between EI and gait speed (9–11, 29, 30, 32–34, 37). The combined effect size for EI and gait speed was *r* = −0.01 (95% CI: −0.07 to −0.06, *P* = 0.67, *I*^2^ = 58.00), indicating no linear correlation between the two with moderate heterogeneity. Subgroup analysis showed a weak negative correlation between usual gait speed (UGS) and EI (*r* = −0.17, 95% CI: −0.27 to −0.07, *P* < 0.001, *I*^2^ = 0.000), while there was a weak positive correlation between maximal gait speed (MGS) and EI (*r* = 0.10, 95% CI: 0.01 to 0.18, *P* = 0.018, *I*^2^ = 55.82) (Figure 5). There was no indication of publication bias (*t* = 0.70, *p* = 0.49; Supplementary Figure 3A).

Ten studies (involving 1,523 participants) investigated the association between MT and gait speed (9, 11, 23, 26, 28, 30, 34, 35, 38, 39). The combined effect size for MT and gait speed was *r* = −0.08 (95% CI: −0.12 to −0.04, *P* = 0.78, *I*^2^ = 69.01), indicating no linear correlation between the two with moderate heterogeneity. Subgroup analysis showed a weak negative correlation between UGS and MGS with MT (*r* = −0.09, 95% CI: −0.15 to −0.03, *P* = 0.002, *I*^2^ = 0.000) (*r* = −0.08, 95% CI: −0.13 to −0.02, *P* < 0.001, *I*^2^ = 0.000) (Figure 3). Due to indication of publication bias (*t* = 2.01, *p* = 0.03; Supplementary Figure 3B), the Trim and Fill procedure was applied, adjusting the mean effect size to 0.79 (95% CI = −0.11 to 3.33).

Five studies (involving 222 participants) investigated the association between FL and gait speed (23, 26, 30, 32, 38). The combined effect size for FL and gait speed was *r* = −0.03 (95% CI: −0.15 to 0.09, *P* = 0.602, *I*^2^ = 0.000), indicating no linear correlation between the two. Subgroup analysis showed a weak negative correlation between UGS and MGS with FL (*r* = −0.06, 95% CI: −0.27 to 0.14, *P* = 0.528, *I*^2^ = 37.31) (*r* = −0.01, 95% CI: −0.16 to 0.14, *P* = 0.855, *I*^2^ = 0.000) (Figure 5). Moreover, there was no indication of publication bias (*t* = 0.36, *p* = 0.72; Supplementary Figure 3C).

Four studies (involving 123 participants) investigated the association between PA and gait speed (23, 26, 30, 38). The combined effect size for PA and gait speed was *r* = 0.11 (95% CI: −0.05 to 0.27, *P* = 0.94, *I*^2^ = 0.000), indicating weak linear correlation between the two. Subgroup analysis showed a weak correlation between UGS and PA (*r* = 0.24, 95% CI: 0.02 to 0.43, *P* = 0.031, *I*^2^ = 0.000), while there was a weak negative correlation between MGS and PA (*r* = −0.05, 95% CI: −0.29 to 0.19, *P* = 0.674, *I*^2^ = 0.000) (Figure 5). Across studies, no indication of publication bias (*t* = 0.19, *p* = 0.85; Supplementary Figure 3D) was observed.

#### 3.3.2 Chair stand test

**FIGURE 6:**
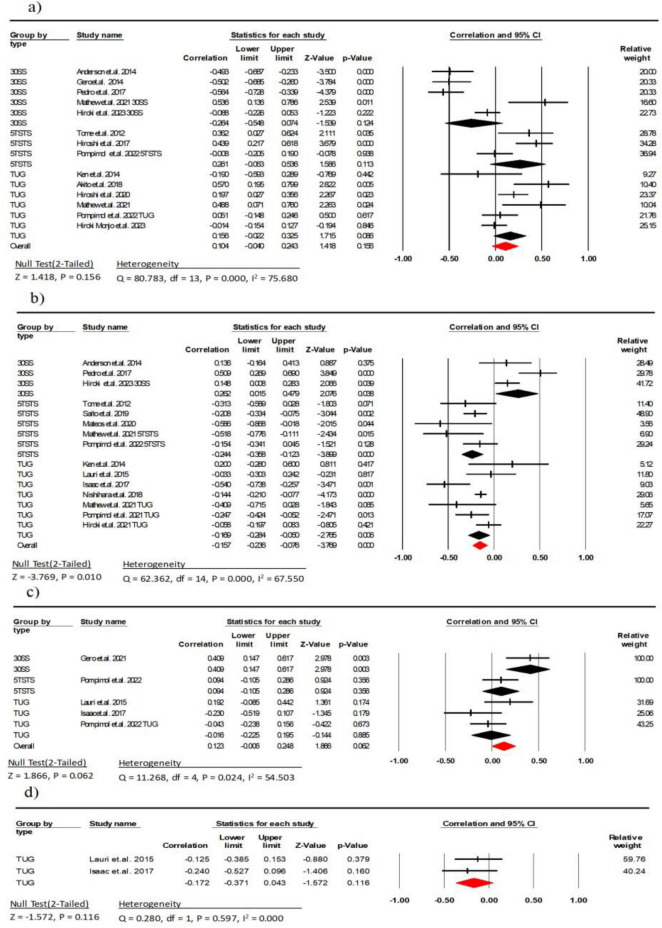
Associations (*r*_*z*_ values) between chair stand test and **(a)** echo intensity, **(b)** muscle thickness **(c)** fascicle length, **(d)** pennation angle. 30SS, 30-s Sit-to-Stand; 5TSTS, 5-time Sit-To-Stand; TUG, Timed Up-and-Go; CI, confidence interval; df, degrees of freedom.

Eleven studies (involving 931 participants) investigated the association between EI and the chair stand test (9–12, 32–34, 37, 39–41). The combined effect size of EI and chair stand test was *r* = 0.10 (95% CI: −0.04 to 0.24, *P* = 0.15, *I*^2^ = 75.68), with a weak statistical correlation and considerable heterogeneity. Subgroup analyses showed a weak negative correlation between EI and the 5TSTS and the 30SS (*r* = −0.26, 95% CI: −0.06 to 0.53, *P* = 0.11, *I*^2^ = 69.17; *r* = −0.26, 95% CI: −0.54 to 0.07, *P* = 0.12, *I*^2^ = 7.84, respectively). There was a weak correlation between EI and the TUG (*r* = 0.15, 95% CI: −0.02 to 0.32, *P* = 0.08, *I*^2^ = 55.39) (Figure 6). The Begg and Mazumdar rank correlation (*t* = 0.51, *p* = 0.61) and the symmetrical funnel plot (Supplementary Figure 4A) suggest publication bias was absent.

Twelve studies (involving 1,605 participants) investigated the association between MT and the chair stand test (11–14, 25, 30, 33, 34, 36, 39, 41, 42). The combined effect size of MT and chair stand test was *r* = −0.15 (95% CI: −0.23 to −0.07, *P* < 0.001, *I*^2^ = 67.55), with a weak statistical correlation and moderate heterogeneity. Subgroup analyses showed a moderate negative weak between MT and the 5TSTS and the TUG (*r* = −0.24, 95% CI: −0.35 to −0.12, *P* < 0.001, *I*^2^ = 15.85; *r* = −0.16, 95% CI: −0.28 to −0.05, *P* = 0.006, *I*^2^ = 55.75, respectively). There was a weak correlation between MT and the 30SS (*r* = 0.26, 95% CI: 0.01 to 0.47, *P* = 0.03, *I*^2^ = 60.27) (Figure 6). There was no indication of publication bias (*t* = 0.22, *p* = 0.82; Supplementary Figure 4B).

Tour studies (involving 237 participants) investigated the association between FL and the chair stand test (12, 26, 32, 38). The combined effect size of FL and chair stand test was *r* = 0.12 (95% CI: −0.01 to 0.24, *P* = 0.06, *I*^2^ = 54.50), with a weak statistical correlation and moderate heterogeneity. Subgroup analysis showed a moderate correlation between FL and the 30SS (*r* = 0.40, 95% CI: −0.14 to 0.61, *P* = 0.003, *I*^2^ = 0.000), and a weak correlation with the 5TSTS (*r* = 0.09, 95% CI: −0.10 to 0.28, *P* = 0.356, *I*^2^ = 0.000). There was a weak correlation between FL and the TUG (*r* = −0.01, 95% CI: −0.22 to 0.19, *P* = 0.02, *I*^2^ = 47.49) (Figure 6). There was also no indication of publication bias (*t* = 0.20, *p* = 0.85; Supplementary Figure 4C).

Two studies (involving 88 participants) investigated the association between PA and the TUG (26, 38). The combined effect size of EI and chair stand test was *r* = −0.17 (95% CI: −0.37 to 0.04, *P* = 0.11, *I*^2^ = 0.000), with a weak statistical correlation (Figure 6).

## 4 Discussion

The objective of this meta-analysis was to comprehensively examine the association between US parameters, muscle strength, and sarcopenia-related exercise performance in healthy older adults. The results of the meta-analysis indicate moderate to strong correlations between EI and MT with maximal strength, explosive power, and handgrip strength in the context of muscle strength. Additionally, CSA shows a strong correlation with maximal strength. However, regarding sarcopenia-related exercise performance, no significant correlations emerged between US parameters and gait speed. The strength of association with sit-to-stand tests varied based on the specific test type, with EI and MT demonstrating only weak correlations in these tests. Furthermore, none of the US parameters demonstrated a significant correlation with TUG test.

### 4.1 Correlation between US parameters and muscle strength

According to our study findings, among older individuals, EI, MT, and CSA appear to be robust indicators for detecting maximal muscle strength. We observed a moderate negative correlation (*r* = −0.56) between EI and maximal muscle strength, consistent with previous studies (24). This suggests that as individuals age, non-muscular components such as fat and connective tissue gradually increase in the muscle, leading to higher EI in US images. Supporting this, existing literature recognizes EI as an effective indicator reflecting the content of non-muscular components in muscle tissue (43). Additionally, a moderate to strong positive correlation was noted between MT (*r* = 0.43) and CSA (*r* = 0.67) with maximal muscle strength. Older adults commonly undergo a decline in muscle quality and size, possibly attributable to a reduction in the number and diameter of muscle fibers (44). This is consistent with the decrease in MT and CSA, affecting overall muscle strength. However, the correlation with PA and muscle strength did not reach significance. This lack of significance may be attributed to the relatively minor impact of PA in older individuals, exhibiting greater variability compared to other muscle parameters. Additionally, technical factors, including US image resolution and sampling position, might influence the measurement of PA, introducing some uncertainty. Past research on the relationship between PA and muscle strength has produced inconsistent results, potentially influenced by individual differences, measurement methods, and study designs (45, 46).

Our research findings highlight that only two US parameters, specifically EI and MT, demonstrate significant correlations with explosive force and handgrip strength. Notably, we identified a substantial negative correlation between EI and explosive force (*r* = −0.47). Increased EI is commonly associated with a heightened presence of non-muscular components, such as fat and connective tissue, within the muscle (43). These non-muscular components may adversely impact muscle elasticity and power transmission, leading to a reduction in explosive force. Consequently, the rise in US EI could be attributed to various factors linked to intramuscular fat infiltration, encompassing a decline in single fiber contraction speed, decreased power output, modifications in muscle mechanical characteristics, heightened muscle stiffness, and alterations in fiber shortening and expansion (47, 48). In contrast, MT exhibits a substantial positive correlation with explosive force (*r* = 0.53). This discovery aligns with prior research supporting the notion that greater MT corresponds to increased muscle strength (49). Larger MT typically signifies a higher number of muscle fibers, contributing to enhanced explosive force. This association can be explained by the fact that MT directly reflects the augmentation in muscle quality available for power generation (50).

Our research results emphasize the significance of EI and MT as US parameters for assessing handgrip strength. The specific data reveals that the correlation coefficients observed (*r* = −0.32 and *r* = 0.26, respectively) are statistically significant (*p* < 0.05). This serves to underscore the negative correlation between EI and handgrip strength, as well as the positive correlation between MT and handgrip strength. These findings are in accordance with prior research, which recognizes handheld dynamometry as a reliable indicator of muscle strength (51). They contribute to a more profound comprehension of the relationship between muscle structure and function, providing practical implications for the clinical evaluation of handgrip strength.

### 4.2 Correlation between US parameters and sarcopenia-related exercise performance

US parameters have been a subject of considerable attention in gait speed research. However, our data reveals a notably weak correlation, approaching nonexistence, between these US parameters and both UGS and MGS (*r* = −0.01 to *r* = 0.11, respectively). Consistent with a previous study involving a cohort of healthy adults, US parameters showed no significant association with gait speed (*r* = −0.02 to *r* = 0.14) (40). The main contributing factor for this disparity could be the restricted age range of the participants. The study by Mangine et al. (52) emphasized that within the younger demographic, specific US parameters may demonstrate some correlation with gait speed. However, this correlation is unstable and marked by notable individual variations. Furthermore, the study indicates that following the correction for subcutaneous fat, US parameters show a notably enhanced correlation with gait speed in older adults (53). Hence, consideration of age range and conducting subcutaneous fat correction are crucial factors for a thorough comprehension of the association between US parameters and gait speed.

We conducted a study on the relationship between US parameters and chair stand tests, finding a weak correlation and significant heterogeneity among the parameters. Subgroup analysis revealed correlations between EI, MT, and FL with the 30SS (*r* = −0.26, *r* = 0.26, and *r* = 0.41, respectively). Notably, the association between FL and leg flexibility was more pronounced, which is unsurprising considering that the chair stand test occurs within a typical range of motion and is relatively straightforward. Our findings align with prior reports, (54) suggesting that releasing the fascia of leg muscles contributes to enhanced mobility by expanding the range of motion in the hip and knee joints. However, we observed no significant correlation between the TUG test and the various parameters. Given our emphasis on thigh muscles and the absence of observed correlations between the parameters and TUG, in contrast to previous findings on the contribution of the gastrocnemius and soleus muscles to TUG (34), we assert that further in-depth research is needed to ascertain the value of muscle US parameters in predicting functional physical fitness in older adults. The TUG test encompasses diverse rapid movements, including walking and turning, implying that its complexity may warrant a more comprehensive examination.

Through the synthesis of findings from two studies, it has been established that parameters derived from non-invasive and radiation-free US can serve as predictive indicators for muscle strength and physical function in older adults. This comprehensive understanding encompasses multiple facets of US application in assessing the muscle health of older individuals, covering structural parameters such as MT and CSA, in addition to EI. However, it is imperative to acknowledge that obtaining dependable data from US measurements necessitates precise methodologies. Variables including the patient’s positioning, muscle contraction during measurement, and the angle of the sensor relative to the skin surface all have the potential to influence muscle US measurements.

This study has several limitations. Firstly, the meta-analysis was constrained to cross-sectional design studies, limiting the exploration to associations and precluding the establishment of causal relationships. Secondly, insufficient data for the calf impede a comprehensive understanding of lower limb walking and gait-related aspects. Secondly, due to a lack of sufficient data for the calf, our understanding of lower limb walking and gait-related aspects is not comprehensive. Additionally, some included studies lacked effective control for confounding variables. The focus on outcomes in older adults did not involve a separate investigation of gender, introducing uncertainties about potential gender differences. Finally, the presence of heterogeneity in chair test evaluations stems from the utilization of diverse methodologies, underscoring the importance of meticulously selecting suitable approaches to mitigate pertinent limitations when investigating the correlation with chair stand test performance.

To address the aforementioned limitations and advance future research in this field, combining ultrasound with complementary techniques such as bioimpedance analysis (BIA) and functional near-infrared spectroscopy (fNIRS) could provide a more comprehensive evaluation of muscle health in older adults. BIA offers non-invasive assessment of body composition, including muscle mass and fat distribution, (55) which could provide additional insights into the relationship between muscle quality and overall body composition when used alongside ultrasound. Similarly, fNIRS methodology allows for real-time monitoring of muscle oxygenation and hemodynamics during exercise, (56) potentially bridging the gap between structural measurements and functional outcomes, particularly in areas where our analysis showed weak correlations, such as gait speed and TUG tests. This multi-modal approach could lead to improved strategies for early detection and monitoring of sarcopenia and more targeted interventions, ultimately enhancing our understanding of muscle function and quality of life in aging populations.

## 5 Conclusion

This meta-analysis reveals significant correlations between ultrasound parameters (echo intensity and muscle thickness) and muscle function in healthy older adults. These findings underscore the potential of ultrasound as a diagnostic tool for assessing muscle characteristics in this population. However, the observed heterogeneity across studies highlights the need for further research. Future investigations should focus on standardizing measurement techniques, establishing age- and sex-specific reference values, and conducting longitudinal studies with larger sample sizes. Such efforts could enhance the clinical applicability of ultrasound in assessing and monitoring muscle health in older adults, potentially improving early detection and intervention strategies for age-related muscle changes.

## Data Availability

The raw data supporting the conclusions of this article will be made available by the authors, without undue reservation.
