# Supplementary material for: Exploring the relationship between ultrasound parameters and muscle strength in older adults: a meta-analysis of sarcopenia-related exercise performance
Source: Front Med (Lausanne). 2024 Sep 27;11:1429530. doi: 10.3389/fmed.2024.1429530 (PMC11466788; doi:10.3389/fmed.2024.1429530)
Supplement: Supplementary file 1 [file Data_Sheet_1.docx]

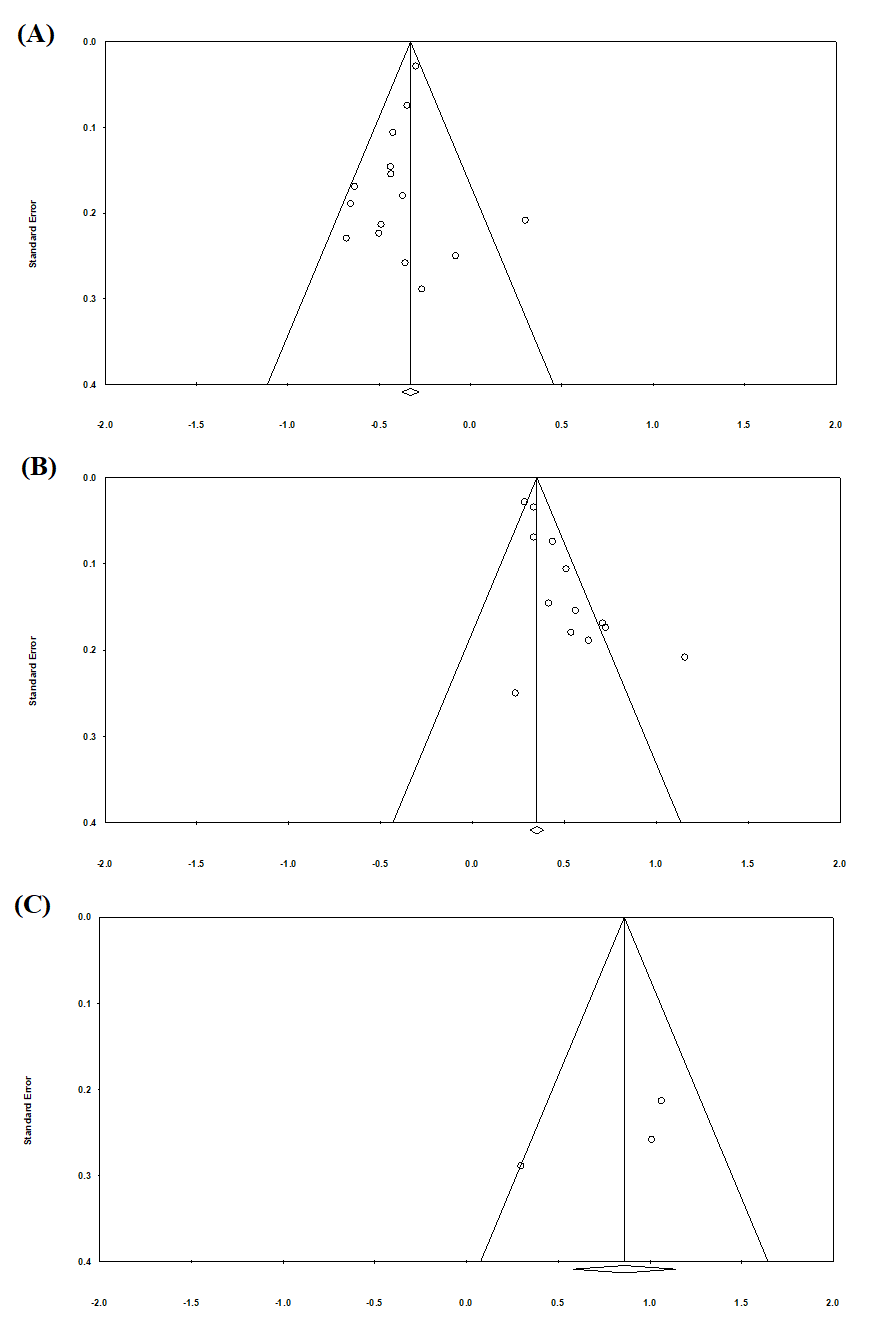


**Fig S1.** Maximal strength and (A) Echo intensity, (B) Muscle thickness, (C) Cross-sectional area: funnel plot of standard errors plotted against study effect sizes.


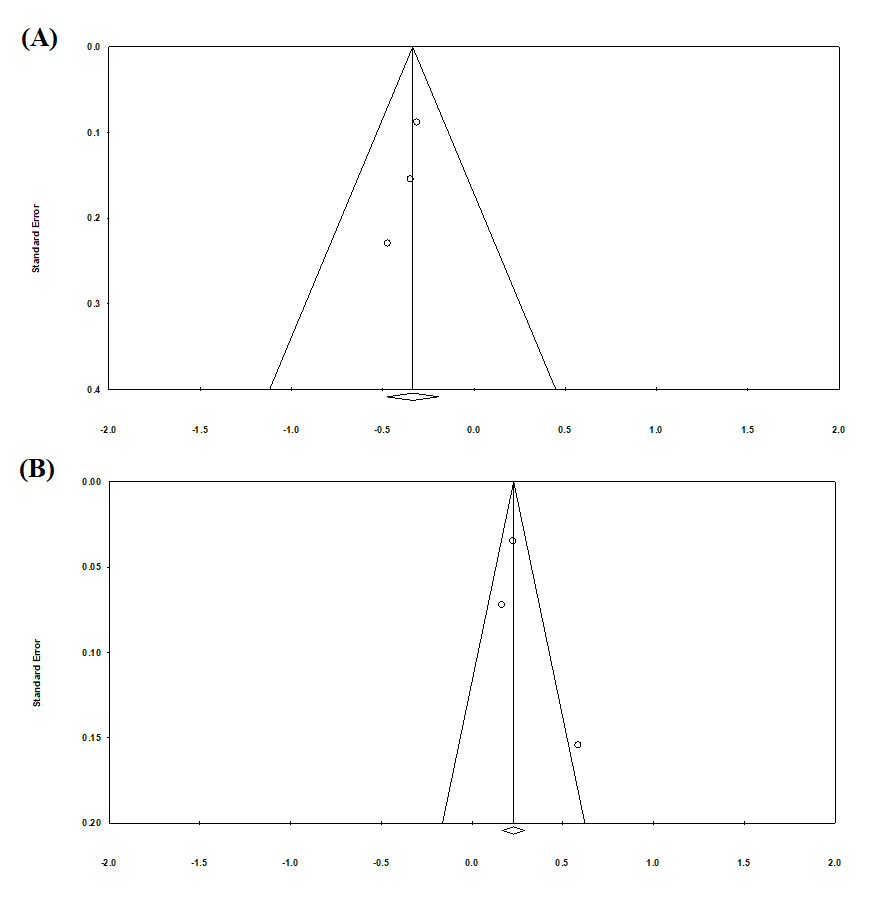


**Fig S2.** Handgrip strength and (A) Echo intensity, (B) Muscle thickness: funnel plot of standard errors plotted against study effect sizes.


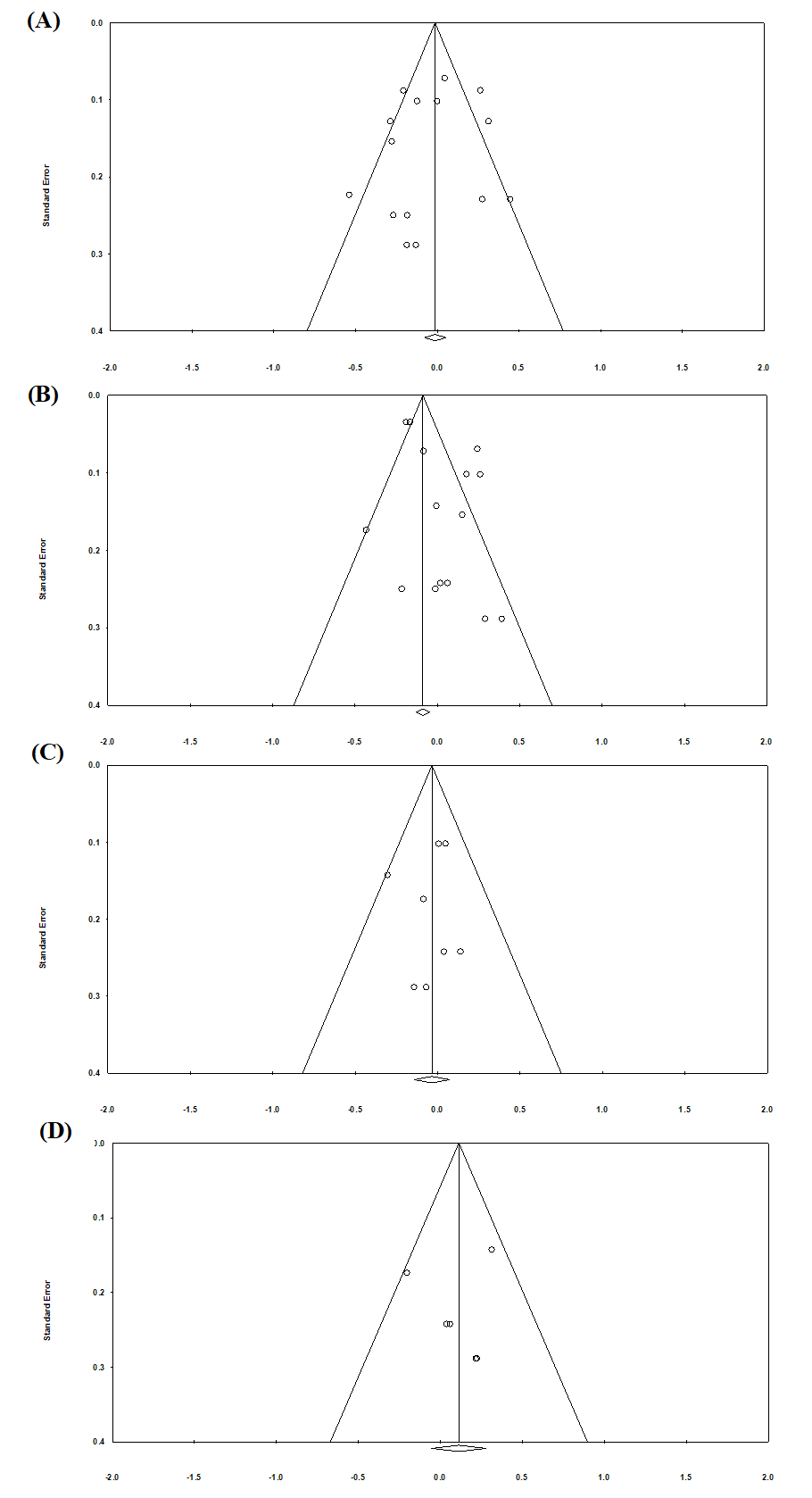


**Fig S3.** Gait speed and (A) Echo intensity, (B) Muscle thickness; (C) Cross-sectional area, (D) Pennation angle: Funnel plot of standard errors plotted against study effect sizes.


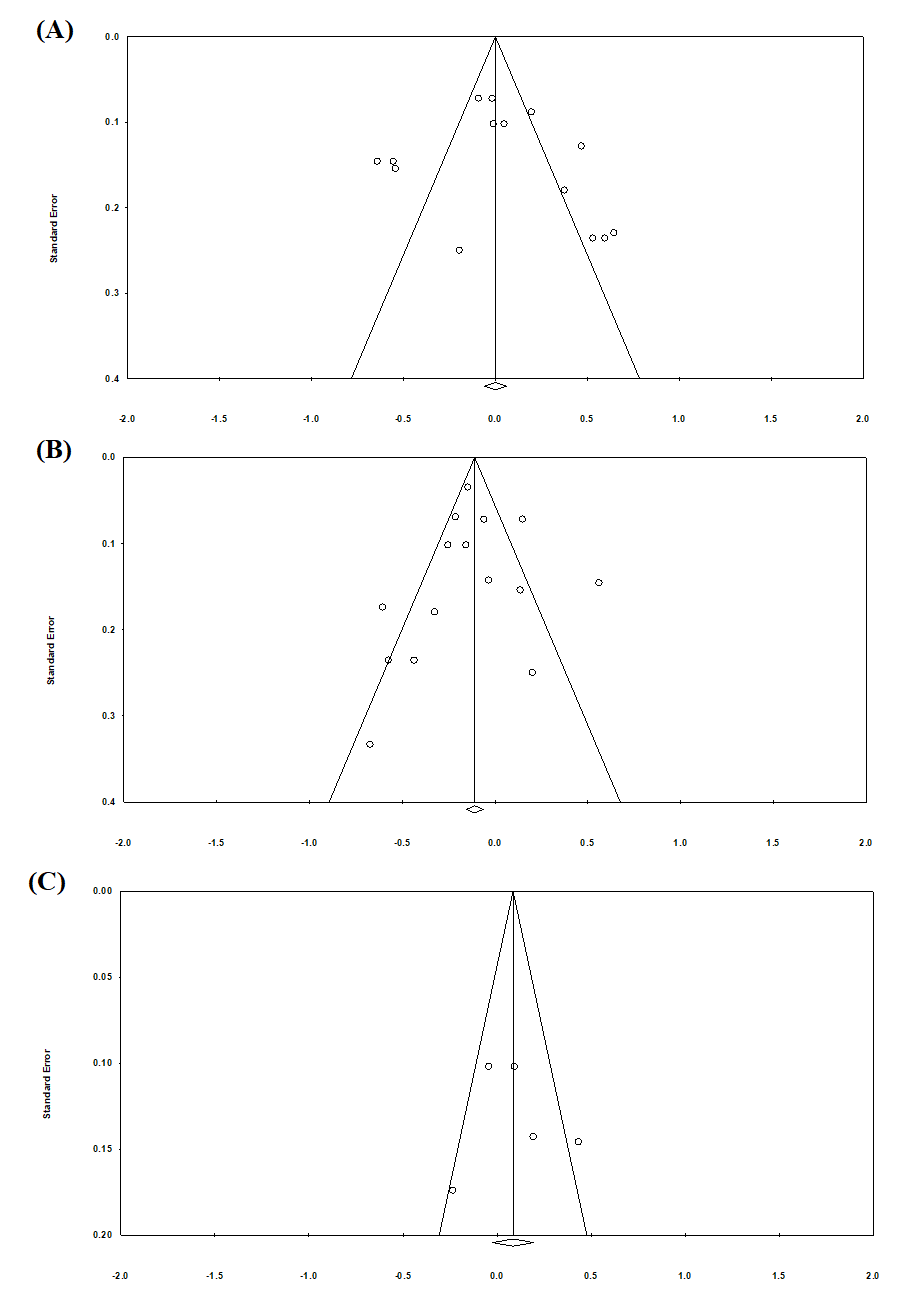


**Fig S4.** Chair stand test and (A) Echo intensity, (B) Muscle thickness (C) Fascicle length: Funnel plot of standard errors plotted against study effect sizes.
