# Supplementary material for: Exploring the relationship between ultrasound parameters and muscle strength in older adults: a meta-analysis of sarcopenia-related exercise performance
Source: Front Med (Lausanne). 2024 Sep 27;11:1429530. doi: 10.3389/fmed.2024.1429530 (PMC11466788; doi:10.3389/fmed.2024.1429530)
Supplement: Supplementary file 2 [file Table_1.DOCX]

| **Strategy** | **((#1 AND #2 AND #3 AND #4 AND #5 AND) NOT (‘disease OR ‘patients’)) limited to ‘Humans’ & ‘English’** | |
| --- | --- | --- |
|  | Subject words | Free words |
| **#1** | Aged | “Elderly OR “Old” OR “age” OR “old age” OR “older adult*” OR “older people” OR elder* OR “aging adults” OR “older person*” OR “old-age” OR Older OR “elderly person*” OR Aging OR Ageing OR Aged OR “old man” OR “old men” OR “old women” OR “old woman” |
| **#2** | Ultrasonography | “Ultrasound Imaging” OR “Ultrasound Parameters” OR “Echo intensity” OR “EI” OR “echo” “OR “muscle thickness” OR “MT” OR “fascicle length” “FL” OR “PA” OR “pennation angle” OR “cross-sectional area” OR “CSA” |
| **#3** | Thigh | Thighs OR “Quadriceps Muscle” OR “Quadriceps Muscles” OR “Quadriceps Femoris” OR “Vastus Medialis” OR “Vastus Intermedius” OR “Rectus Femoris” OR “Vastus Lateralis” OR “knee extensors” OR Knee OR “Hamstring Muscles” OR “Hamstring Muscle” OR “Biceps Femoris” OR “Semimembranosus” OR “knee flexors” |
| **#4** | Muscle Strength | Muscle Strength OR “Physical Functional Performance” OR “Muscle Strength Dynamometer” OR Strength OR “maximal voluntary contraction” OR “Maximal voluntary isometric contraction” OR Power OR Force OR Torque OR MVC OR MVIC OR “explosive force” OR “explosive strength” OR “explosive torque” OR “Rate force development” OR RFD OR RTD OR “knee extensor*” OR “knee flexor*” OR “ankle dorsiflexor*” OR “ankle plantar flex* OR “Muscle Strength*” OR “sarcopenia-related exercise performance” OR “sarcopenia” OR “muscle atroph*” OR “muscular atroph”*OR “Physical Functional Performance” OR “Physical Functional Performance*” OR “Physical Performance*” OR “Functional Performance*” OR “Physical Function*” OR “muscle function” OR “neuromuscular function” OR “Hand Strength” OR “Hand Grip Strength” OR “grip strength OR Grip*” OR HGS OR “Gait Analysis” OR Gait OR Gaits OR “Gait Speed*” OR “Walking Speed*” OR “Walking Pace*” OR TUG OR “Walk Test OR walk*” OR “chair stand time” OR “sit-to-stand” OR “chair stand” OR “chair stand test” OR CST OR stand* |
|  | Physical Functional Performance |  |
| **#5** | Correlation | “Correlate*” OR “Associat*” OR “Relat*” |

Search strategy
