# Supplementary material for: Exploring the relationship between ultrasound parameters and muscle strength in older adults: a meta-analysis of sarcopenia-related exercise performance
Source: Front Med (Lausanne). 2024 Sep 27;11:1429530. doi: 10.3389/fmed.2024.1429530 (PMC11466788; doi:10.3389/fmed.2024.1429530)
Supplement: Supplementary file 3 [file Table_2.DOCX]

| NO. | Title | Clear inclusion criteria | Study subjects/ setting described | Valid exposure measure | Standard condition | Confounders identified | Confounders minimised | Valid outcome measure | Appropriate statistical analysis | Total |
| --- | --- | --- | --- | --- | --- | --- | --- | --- | --- | --- |
| 1 | Eduardo et.al. 2012 | Yes | Unclear | Yes | Yes | Unclear | Unclear | Yes | Yes | 5/8 |
| 2 | Fukumoto et.al. 2012 | Yes | Yes | Yes | Yes | Yes | Yes | Yes | Yes | 8/8 |
| 3 | Tome et.al. 2012 | Yes | Yes | Yes | Yes | Unclear | Unclear | Yes | Yes | 6/8 |
| 4 | Sabrina et.al. 2012 | Yes | Yes | Yes | Yes | Yes | Unclear | Yes | Yes | 7/8 |
| 5 | Gero et.al. 2013 | Yes | Yes | Yes | Yes | Yes | Yes | Yes | Yes | 5/8 |
| 6 | Yuya et.al. 2013 | Yes | Yes | Yes | Yes | Yes | Yes | Yes | Yes | 8/8 |
| 7 | Ken et.al. 2014 | Yes | Unclear | Yes | Yes | Unclear | Unclear | Yes | Yes | 5/8 |
| 8 | Anderson et.al. 2014 | Yes | Unclear | Yes | Yes | Unclear | Unclear | Yes | Yes | 5/8 |
| 9 | Gero et.al. 2014 | Yes | Yes | Yes | Yes | Unclear | Unclear | Yes | Yes | 6/8 |
| 10 | Lauri et.al. 2015 | Yes | Yes | Yes | Yes | Unclear | Unclear | Yes | Yes | 6/8 |
| 11 | Hiroshi et.al. 2017 | Yes | Yes | Yes | Yes | Yes | Yes | Yes | Yes | 8/8 |
| 12 | Pedro et.al. 2017 | Yes | Yes | Yes | Yes | Yes | Yes | Yes | Yes | 8/8 |
| 13 | Isaac et.al. 2017 | Yes | Yes | Yes | Yes | Yes | Yes | Yes | Yes | 8/8 |
| 14 | Hisashi et.al. 2018 | Yes | Yes | Yes | Yes | Yes | Yes | Yes | Yes | 8/8 |
| 15 | Mitchel et.al. 2018 | Yes | Yes | Yes | Yes | Yes | Yes | Yes | Yes | 8/8 |
| 16 | Nishihara et.al. 2018 | Yes | Yes | Yes | Yes | Unclear | Yes | Yes | Yes | 7/8 |
| 17 | Matt et.al. 2018 | Yes | Yes | Yes | Yes | Yes | Yes | Yes | Yes | 8/8 |
| 18 | Akito et.al. 2018 | Yes | Unclear | Yes | Yes | Unclear | Yes | Yes | Yes | 6/8 |
| 19 | Eliane et.al. 2019 | Yes | Unclear | Yes | Yes | Yes | Yes | Yes | Yes | 7/8 |
| 20 | Saito et.al. 2019 | Yes | Yes | Yes | Yes | Yes | Yes | Yes | Yes | 8/8 |
| 21 | Hiroshi et.al. 2020 | Yes | Yes | Yes | Yes | Yes | Yes | Yes | Yes | 8/8 |
| 22 | Akash et.al. 2020 | Yes | Yes | Yes | Yes | Yes | Yes | Yes | Yes | 8/8 |
| 23 | Angulo et.al. 2020 | Yes | Yes | Yes | Yes | Yes | Yes | Yes | Yes | 8/8 |
| 24 | Ahalee et.al. 2021 | Yes | Yes | Yes | Yes | Yes | Yes | Yes | Yes | 8/8 |
| 25 | Mathew et.al. 2021 | Yes | Unclear | Yes | Yes | Yes | Yes | Yes | Yes | 7/8 |
| 26 | Pornpimol et.al. 2022 | Yes | Yes | Yes | Yes | Unclear | Unclear | Yes | Yes | 6/8 |
| 27 | Akito et.al. 2022 | Yes | Unclear | Yes | Yes | Unclear | Unclear | Yes | Yes | 5/8 |
| 28 | Hiroki et.al. 2023 | Yes | Yes | Yes | Yes | Yes | Yes | Yes | Yes | 8/8 |

The Joanna Briggs Institute (JBI) Analytic Cross-Sectional Study Quality Checklist
