# Supplementary material for: Exploring the relationship between ultrasound parameters and muscle strength in older adults: a meta-analysis of sarcopenia-related exercise performance
Source: Front Med (Lausanne). 2024 Sep 27;11:1429530. doi: 10.3389/fmed.2024.1429530 (PMC11466788; doi:10.3389/fmed.2024.1429530)
Supplement: Supplementary file 4 [file Table_3.DOCX]

Characteristics of Studies Included and Measured Activities Related to Muscle Quality, Muscle Strength, and Sarcopenia-Related Exercise Performance

| **Study, year** | **Sample size (sex), age** | **Ultrasound Parameter Outcome Measures/**  **Device Used/ Position Tested** | **Muscle strength parameter/outcome** | **sarcopenia-related exercise performance parameter/outcome** |
| --- | --- | --- | --- | --- |
| Monjo et.al, 2023 | n=195 (M61, F134)  Age: 75.9±5.65yr | EI, MT (LOGIQ e; GE Healthcare, Chicago, IL)/supine/QF | CMJ  Handgrip strength | MGS(m/s)-10m walk  Chair stand test(s)-30SS, TUG |
| Yoshiko, 2022 | n=38 (22, F16)  Age: 71.6 ± 5.1yr | EI, MT (LOGIQ e Premium Ultrasound system; GE Healthcare Japan, Tokyo, Japan)/ supine/RF, VL | MVC: knee extensors | Chair stand test(s)-30SS |
| Muanjai et.al, 2022 | n=99 (M)  Age: 66.6 ± 4.6yr | EI, MT, CSA (M5 series, Shenzhen, Mindray Bio-Medical, China) supine, dominant leg/VL |  | UGS(m/s)-10m walk  MGS(m/s)-6min walk  Chair stand test(s)- 5TSTS, TUG |
| Hill et.al, 2021 | n=21 (M12, F9)  Age: 69.9 ± 4.3yr | EI, MT (LOGIQ Book XP, General Electric, Bedford, UK) Seated/VL |  | Chair stand test(s)- 5TSTS, TUG |
| Farrow et.al, 2021 | n=15(M)  Age: 73±4yr | EI, MT, CSA (GE Logiq S8; GE Healthcare, Milwaukee, WI)/ supine, right leg/RF | MVC: knee extensors  RTD: knee extensors |  |
| Angulo et.al, 2020 | n=12 (M9, F3)  Age: 86 ± 7yr | MT (Esaote MyLab One) /Seated RF | Handgrip strength | Chair stand test(s)- 5TSTS |
| Bali et.al, 2020 | n=25 (M10, F15)  Age: 71.5 ± 5.5yr | EI, CSA (GE Logiq e BT12, GE Healthcare, Milwaukee, WI, USA)/ supine/RF, VL | MVC: knee extensors |  |
| Akima et.al, 2020 | n=132 (M62, F70)  Age: 74.15±2.8yr | EI, (LOGIQ e; GE Healthcare, Chicago, IL, USA) / supine, right leg/QF | Handgrip strength | UGS(m/s)-10min walk  MGS(m/s)-5m walk  Chair stand test(s)- 30SS |
| Saito et.al, 2019 | n=211 (F)  Age: 73.4 ± 6.0yr | MT (Noblus; Hitachi, Tokyo, Japan) Supine, dominant leg/RF | MVC: knee extensors | MGS(m/s)-5m walk  Chair stand test(s)-TUG |
| Guadagnin et.al, 2019 | n=15 (M)  Age: 75.4 ± 5yr | EI, MT, FL, PA (MyLab30 Gold, Esaote Inc., USA)/RF, VL | MVC: knee extensors/flexors | MGS(m/s)-8m walk  UGS(m/s)-8m walk |
| Akito Yoshiko et .al, 2018 | n=22 (M)  Age: 78 ± 8yr | EI (LOGIQ e, GE Healthcare, Duluth, GA, USA)/ supine, right leg/RF | MVC: knee extensors  Handgrip strength | MGS(m/s)-5m walk  UGS(m/s)-5m walk  Chair stand test(s)- 30SS, TUG |
| Matt S. Stock et .al, 2018 | n=23 (M11, F12)  Age: 72yr | EI (GE Logiq e BT12, GE Healthcare, Milwaukee, WI, USA) /supine, right leg/RF | MVC: knee extensors  RTD: knee extensors | MGS (m/s)-10m walk  UGS(m/s)-400m walk  Chair stand test(s)-TUG |
| K Nishihara et. al, 2018 | n=831(F356, M475)  Age: 72.0yr | MT (Mysono U6 Medison, Seoul, Korea)/supine, right leg/RF | MVC: knee extensors  Handgrip strength | MGS(m/s)-8m walk  UGS(m/s)-8m walk |
| Mitchel A et.al, 2018 | n=18 (M)  Age: 74.87 ± 5.83yr | EI, CSA (GE Logiq131 S8, Milwaukee, WI, USA)/supine, right leg/RF | MVC: knee extensors |  |
| Hisashi Kawai et.al, 2018 | n=1239 (M511, F728) | EI, MT (Miru-Cube, Global Health, Kanagawa, Japan)/supine, right leg/QF | MVC: knee extensors/flexors | Chair stand test(s)-30SS |
| Isaac Selva Raj et.al, 2017 | n=36 (M20, F16)  Age: 68.2 ± 5.3yr | MT, FL, PA (Miru-Cube, Global Health, Kanagawa, Japan)/ Seated, right leg/QF | MVC: knee extensors  RTD: knee extensors | MGS(m/s)-5m walk  Chair stand test(s)-TUG |
| Pedro Lopez BSc et.al, 2017 | n=52 (M26, F26)  Age: 74.8±3.3yr | EI, MT (Nemio XGToshiba, Japan)/ Supine/QF | MVC: knee extensors  RTD: knee extensors | Chair stand test(s)-30SS |
| Hiroshi Akima et.al, 2017 | n=50 (M)  Age: 66 ± 5.4yr | EI (LOGIQ e; GE Healthcare, Chicago, IL, USA)/Supine/QF |  | MGS(m/s)-5m walk  UGS(m/s)-6min walk  Chair stand test(s)- 30SS |
| Lauri Stenroth et.al, 2015 | n=52 (M26, F26)  Age: 74.8±3.3yr | MT, FL, PA (LOGIQ e, GE Healthcare, Duluth, GA, USA)/ Supine/QF | MVC: knee extensors | UGS(m/s)-6min walk  Chair stand test(s)-TUG |
| GeroScience et.al, 2014 | n=50 (M)  Age: 66.1 ± 4.5yr | EI, MT (Nemio XG Toshiba, Japan)/ Supine/QF | MVC: knee extensors  RTD: knee extensors  CMJ | Chair stand test(s)- 30SS |
| Anderson Rech et.al, 2014 | n=45 (F)  Age: 70.28 ± 6.2yr | EI, MT (Nemio XG Toshiba, Japan)/Supine/QF | RTD: knee extensors  Handgrip strength | UGS(m/s)-6m walk  Chair stand test(s)-30SS |
| Ken Nishihara et.al, 2014 | n=19 (M14, F5)  Age: 73±3.2yr | EI, MT (Mysono U6; Samsung Medison, Seoul, Korea)/ Supine/QF | MVC: knee extensors | MGS(m/s)-5m walk  UGS(m/s)-6m walk  Chair stand test(s)- TUG |
| Yuya et.al, 2013 | n=184 (M)  Age: 74.4 ± 5.9yr | EI, MT (SonoSite 180 Plus; SonoSite Japan, Tokyo, Japan)/ Seated/QF | MVC: knee extensors |  |
| GeroScience et.al, 2013 | n=26 (M)  Age: 67.8 ± 4.8yr | EI, MT, PA (LOGIQ e; GE Healthcare UK Ltd, Chalfont, Buckinghamshire, England)/Supine, right leg/QF | MVC: knee extensors |  |
| Lee et.al, 2012 | n=20 (M)  Age: 75.5±5.6yr | MT, FL, PA (Aloka 1100)/ Supine/GM |  | MGS(m/s)-4m walk  UGS(m/s)-6min walk |
| Ikezoe et.al, 2012 | n=34 (F)  Age: 84.2yr | EI, MT (LOGIQ Book Xp, GE Healthcare Japan, Tokyo, Japan)/ Supine, right leg/RF, VL | MVC: knee extensors | Chair stand test(s)-TUG |
| Fukumoto et.al,2012 | n=92 (F)  Age: 70.4 ± 5.5yr | EI, MT (LOGIQ e；GE Healthcare UK Ltd.，Chalfont，Buckinghamshire，England)/Seated/QF | MVC: knee extensors |  |
| Eduardo et.al, 2012 | n=31 (M)  Age: 65.5±5.0yr | EI, MT (Philips, VMI, MG, Brazil)/ Supine/RF | MVC: knee extensors |  |
| M, male; F, female; EI, echo intensity; MT, muscle thickness; FL, fascicle length; PA, pennation angle; CSA, cross-sectional area; MVC, maximum voluntary contraction; RTD, rate of torque development; RFD, rate of force development; CMJ, counter movement jump; VL, vastus lateralis; RF, rectus femoris; QF, quadriceps femoris; GM, gastrocnemius muscle; MGS, Maximal Gait Speed; UGS, Usual Gait Speed; 30SS, 30-second Sit-to-Stand; 5TSTS, 5-time Sit-To-Stand; TUG, Timed Up-and-Go. | | | | |
